# Block-Wise Two-Dimensional Maximum Margin Criterion for Face Recognition

**DOI:** 10.1155/2014/875090

**Published:** 2014-01-22

**Authors:** Xiao-Zhang Liu, Guan Yang

**Affiliations:** ^1^School of Computer Science, Dongguan University of Technology, Dongguan 523808, China; ^2^School of Computer Science, Zhongyuan University of Technology, Zhengzhou 450007, China

## Abstract

Maximum margin criterion (MMC) is a well-known method for feature extraction and dimensionality reduction. However, MMC is based on vector data and fails to exploit local characteristics of image data. In this paper, we propose a two-dimensional generalized framework based on a block-wise approach for MMC, to deal with matrix representation data, that is, images. The proposed method, namely, block-wise two-dimensional maximum margin criterion (B2D-MMC), aims to find local subspace projections using unilateral matrix multiplication in each block set, such that in the subspace a block is close to those belonging to the same class but far from those belonging to different classes. B2D-MMC avoids iterations and alternations as in current bilateral projection based two-dimensional feature extraction techniques by seeking a closed form solution of one-side projection matrix for each block set. Theoretical analysis and experiments on benchmark face databases illustrate that the proposed method is effective and efficient.

## 1. Introduction

Most well-known appearance-based face recognition methods are based on subspace techniques for feature extraction, such as principal component analysis (PCA) [1], linear discriminant analysis (LDA) [2], and maximum margin criterion (MMC) [3]. These conventional appearance-based techniques are based on the so-called vector-space model. Under this model, the original two-dimensional (2D in short) image data are reshaped into a one-dimensional (1D in short) long vector by stacking either rows or columns of the image. This vector-space model makes pattern recognition and analysis techniques be conveniently applied to image domain, and numerous successes have been achieved. However, it also introduces the following problems in practical applications. First, the intrinsic 2D structure of image matrix is removed. As a result, the spatial information stored in the 2D image is discarded and not effectively utilized for representation and recognition. Second, each image sample is modeled as a point in a high-dimensional space; for example, for an image of size 112 × 92, the commonly used image size in face recognition, the dimension of the vector space is 10304, and the size of the scatter matrices is 10304 × 10304. Obviously, a large number of training samples are needed to get a reliable and robust estimation of data statistics. This problem, known as curse of dimensionality, is often confronted in real applications. Third, a very limited number of data are usually available in real applications such that the small sample size (SSS) problem [4] comes forth frequently in practice.

To overcome the above drawbacks, efforts have been made to seek to extract the features directly without vectorization of image samples; that is, the representation of an image sample is retained in matrix form [5]. With this consideration, some bilateral projection based 2D feature extraction techniques have been proposed for seeking transforms on both sides of the image matrix, such as GLRAM (generalized low-rank approximation of matrices) [6], which can be seen as a kind of two-dimensional PCA, and 2DLDA (two dimensional LDA) [7], which can implicitly resolve the SSS problem suffered by LDA. These 2D methods are more computationally efficient than their 1D counterparts, respectively. And, GLRAM and 2DLDA are evaluated empirically to be more effective than PCA and LDA, respectively [6, 7], due to preserving the intrinsic spatial information of data matrix.

Furthermore, two dimensional MMC (2DMMC) has been proposed [8], which aims to find two orthogonal projection matrices to project the original image matrices to a low-dimensional matrix subspace. In the projected subspace, a sample is close to those in the same class but far from those in different classes. Both theoretical analysis and experiments on benchmark face recognition datasets illustrate that 2DMMC is more effective and more efficient than GLRAM and 2DLDA. However, like GLRAM and 2DLDA, the algorithm of 2DMMC involves iterations and alternations of computing two-side projection matrices, which are time-consuming, and an arbitrary initial value before iterations cannot guarantee the global optimum.

In this paper, we propose a novel framework for 2D generalization of conventional MMC to extract discriminating features directly from 2D face images. The proposed algorithm, namely block-wise two-dimensional maximum margin criterion (B2D-MMC), aims to find local subspace projections by obtaining one-side projection matrix in each block set, such that in the subspace a block is close to those belonging to the same class but far from those belonging to different classes. B2D-MMC introduces a block-wise dividing method for face images as in [9], and the dividing method has been proven to be reliable. Based on one-side projection and block-wise learning, B2D-MMC eludes seeking iterative and alternating two projection matrices, as in GLRAM, 2DLDA and 2DMMC, and has more power of learning local characteristics of images.

The rest of this paper is organized as follows.  Section 2 provides background information on 2DMMC. In Section 3, our Block-wise Two Dimensional Maximum Margin Criterion is proposed. The experiments on standard face recognition datasets are demonstrated in Section 4. Finally, we draw our conclusions in Section 5.

## 2. Review on MMC and 2DMMC

### 2.1. LDA and MMC

The most popular unsupervised feature extraction method is principal component analysis (PCA). It aims to find a subspace in which the variance of the projected data is a maximum. But PCA does not take into account the class information, so the features extracted are not very suitable for classification [2]. Linear discriminant analysis (LDA) is a well-known supervised method which has been shown to be more effective than PCA in face recognition tasks [2].

As supervised feature extraction methods, MMC and LDA share the notations of between-class scatter matrix and within-class scatter matrix as follows.

Given a set of *N* sample images {**x**_1_, **x**_2_,…, **x**_*N*_} taking values in the *d*-dimensional vector form, each belonging to one of *C* classes. Assume the *i*th class contains *N*_*i*_ sample vectors **x**_1_^(*i*)^, **x**_2_^(*i*)^,…, **x**_*N*_*i*__^(*i*)^, *i* = 1,2,…, *C*, so *N* = ∑_*i*=1_^*C*^ *N*_*i*_. The mean vector of the *i*th class and that of the sample set are, respectively, given by

(1) $$\begin{array}{c} m_{i}=\frac{1}{N_{i}}\overset{N_{i}}{\underset{j=1}{\sum }}x_{j}^{\left(i\right)},\;\;m=\frac{1}{N}\overset{C}{\underset{i=1}{\sum }}\overset{N_{i}}{\underset{j=1}{\sum }}x_{j}^{\left(i\right)}. \end{array}$$

The between-class scatter matrix **S**_*b*_ and within-class scatter matrix **S**_*w*_ are, respectively, defined as

(2) $$\begin{array}{c} S_{b}=\frac{1}{N}\overset{C}{\underset{i=1}{\sum }}N_{i}\left(m_{i}-m\right){\left(m_{i}-m\right)}^{T},\\ S_{w}=\frac{1}{N}\overset{C}{\underset{i=1}{\sum }}\overset{N_{i}}{\underset{j=1}{\sum }}\left(x_{j}^{\left(i\right)}-m_{i}\right){\left(x_{j}^{\left(i\right)}-m_{i}\right)}^{T}. \end{array}$$

LDA is based on Fisher criterion, which aims to maximize the between-class distance and minimize the within-class distance as follows:

(3) Wopt=argmax⁡W|WTSbW||WTSwW|=[w1,w2,…,wp],

where |·| denotes the determinant of matrix and **w**_*i*_ is the generalized eigenvector of **S**_*b*_ and **S**_*w*_ corresponding to the *i*th largest generalized eigenvalue *λ*_*i*_, that is,

(4) $$\begin{array}{c} S_{b}w_{i}=\lambda_{i}S_{w}w_{i},\;i=1,2,\ldots ,p. \end{array}$$

If **S**_*w*_ is nonsingular, the solution can be obtained by applying an eigendecomposition to matrix **S**_*w*_^−1^ **S**_*b*_. However, in face recognition applications, where generally the number of training images *N* is much smaller than that of pixels in each image *d*, one is confronted with the difficulty that the within-class scatter matrix **S**_*w*_ is always singular [2], since the rank of **S**_*w*_ is at most *N* − *C*. This is so-called the Small Sample Size (SSS) problem which the LDA method suffers from.

As an efficient and robust alternative to LDA, Maximum Margin Criterion (MMC) [3] is defined as

(5) max⁡W tr⁡(WT(Sb−μSw)W),s.t.    WTW=I,

where tr⁡(·) denotes the matrix trace and *μ* is a weighted parameter which is set to  1 in [3]. MMC is to find the optimal projection matrix **W** = [**w**_1_, **w**_2_,…, **w**_*q*_], which is composed of the *q* eigenvectors corresponding to the largest *q* eigenvalues of **S**_*b*_ − *λ* **S**_*w*_.

The constraint **W** ^*T*^ **W** = **I** allows MMC to avoid calculating the inverse of **S**_*w*_ and thus to elude the potential SSS problem.

### 2.2. DLDA and 2DMMC

2DLDA [7] and 2DMMC [8] consider data with matrix representation and share the notations of between-class scatter and within-class scatter as follows.

Let **X**_*j*_^(*i*)^ ∈ ℝ^*n*×*m*^, *j* = 1,2,…, *N*_*i*_, be the images in the sample set belonging to the *i*th class, *i* = 1,2,…, *C*  (*N* = ∑_*i*=1_^*C*^ *N*_*i*_). Both 2DLDA and 2DMMC aim to find two orthogonal projection matrices, **U** ∈ ℝ^*n*×*l*_1_^ and **V** ∈ ℝ^*m*×*l*_2_^, that map each image matrix **X** ∈ ℝ^*n*×*m*^ to **Y** ∈ ℝ^*l*_1_×*l*_2_^, such that **Y** = **U** ^*T*^ **X** **V**. The mean matrix of the *i*th class and that of the sample set are respectively given by

(6) $$\begin{array}{c} M_{i}=\frac{1}{N_{i}}\overset{N_{i}}{\underset{j=1}{\sum }}X_{j}^{\left(i\right)},\;\;M=\frac{1}{N}\overset{C}{\underset{i=1}{\sum }}\overset{N_{i}}{\underset{j=1}{\sum }}X_{j}^{\left(i\right)}. \end{array}$$

In the low dimensional matrix space resulting from the linear transformation **U** and **V**, the between-class scatter ${\tilde{S}}_{b}$ and within-class scatter ${\tilde{S}}_{w}$ are, respectively, defined as

(7) $$\begin{array}{c} {\tilde{S}}_{b}=\mathrm{tr}\left(\overset{C}{\underset{i=1}{\sum }}N_{i}U^{T}\left(M_{i}-M\right)VV^{T}{\left(M_{i}-M\right)}^{T}U\right),\\ {\tilde{S}}_{w}=\mathrm{tr}\left(\overset{C}{\underset{i=1}{\sum }}\overset{N_{i}}{\underset{j=1}{\sum }}U^{T}\left(X_{j}^{\left(i\right)}-M_{i}\right)VV^{T}{\left(X_{j}^{\left(i\right)}-M_{i}\right)}^{T}U\right). \end{array}$$

For both 2DLDA and 2DMMC, the optimal transformations **U** and **V** would maximize ${\tilde{S}}_{b}$ and minimize ${\tilde{S}}_{w}$.

2DLDA proposed in [7] can be formulated as

(8) $$\begin{array}{c} \left(U,V\right)=\arg\underset{U,V}{\max}\frac{{\tilde{S}}_{b}}{{\tilde{S}}_{w}}. \end{array}$$

The optimization (8) is with respect to **U** and **V**, and a closed form solution cannot be obtained.

2DMMC is defined in [8] as

(9) max⁡U,V S~b−μS~w,s.t.  UTU=I,  VTV=I,

where *μ* is a weighted parameter. Also, a closed form solution can not be obtained due to bilateral unknown projections.

Due to the difficulty of computing the optimal **U** and **V** simultaneously, 2DLDA and 2DMMC both utilize iterative alternating schemes; in each iteration, first they optimize the objective with respect to **U** when fixing **V** (**V** is initialized as any orthogonal matrix before iterations) and then optimize the objective with respect to **V** when fixing **U**. The alternating computation framework in each iteration is reviewed below.

*Computation of* **U**. For a fixed **V**, ${\tilde{S}}_{b}$ and ${\tilde{S}}_{w}$ can be rewritten as

(10) $$\begin{array}{c} {\tilde{S}}_{b}=\mathrm{tr}\left(U^{T}S_{b}^{V}U\right),\;\;{\tilde{S}}_{w}=\mathrm{tr}\left(U^{T}S_{w}^{V}U\right), \end{array}$$

where

(11) $$\begin{array}{c} S_{b}^{V}=\overset{C}{\underset{i=1}{\sum }}N_{i}\left(M_{i}-M\right)VV^{T}{\left(M_{i}-M\right)}^{T},\\ S_{w}^{V}=\overset{C}{\underset{i=1}{\sum }}\overset{N_{i}}{\underset{j=1}{\sum }}\left(X_{j}^{\left(i\right)}-M_{i}\right)VV^{T}{\left(X_{j}^{\left(i\right)}-M_{i}\right)}^{T}. \end{array}$$

For 2DLDA, similar to the optimization problem in (3), the optimal **U** can be obtained by computing an eigendecomposition on (**S**_*w*_^**V**^)^−1^ **S**_*b*_^**V**^ that is composed of the *l*_1_ eigenvectors corresponding to the largest *l*_1_ eigenvalues of (**S**_*w*_^**V**^)^−1^ **S**_*b*_^**V**^.

For 2DMMC, similar to the optimization problem in (5), the optimal **U** can be obtained by computing an eigendecomposition on **S**_*b*_^**V**^ − *μ* **S**_*w*_^**V**^, that is composed of the *l*_1_ eigenvectors corresponding to the largest *l*_1_ eigenvalues of **S**_*b*_^**V**^ − *μ* **S**_*w*_^**V**^.

*Computation of* **V**. From the property tr⁡(**A** **A** ^*T*^) = tr⁡(**A** ^*T*^ **A**) for any matrix **A**, when **U** is fixed, a key observation is that ${\tilde{S}}_{b}$ and ${\tilde{S}}_{w}$ can be rewritten as

(12) $$\begin{array}{c} {\tilde{S}}_{b}=\mathrm{tr}\left(V^{T}S_{b}^{U}V\right),\;\;{\tilde{S}}_{w}=\mathrm{tr}\left(V^{T}S_{w}^{U}V\right), \end{array}$$

where

(13) $$\begin{array}{c} S_{b}^{U}=\overset{C}{\underset{i=1}{\sum }}N_{i}{\left(M_{i}-M\right)}^{T}UU^{T}\left(M_{i}-M\right),\\ S_{w}^{U}=\overset{C}{\underset{i=1}{\sum }}\overset{N_{i}}{\underset{j=1}{\sum }}{\left(X_{j}^{\left(i\right)}-M_{i}\right)}^{T}UU^{T}\left(X_{j}^{\left(i\right)}-M_{i}\right). \end{array}$$

For 2DLDA, similar to the optimization problem in (3), the optimal **V** can be obtained by computing an eigendecomposition on (**S**_*w*_^**U**^)^−1^ **S**_*b*_^**U**^ that is composed of the *l*_2_ eigenvectors corresponding to the largest *l*_2_ eigenvalues of (**S**_*w*_^**U**^)^−1^ **S**_*b*_^**U**^.

For 2DMMC, similar to the optimization problem in (5), the optimal **V** can be obtained by computing an eigendecomposition on **S**_*b*_^**U**^ − *μ* **S**_*w*_^**U**^, that is composed of the *l*_2_ eigenvectors corresponding to the largest *l*_2_ eigenvalues of **S**_*b*_^**U**^ − *μ* **S**_*w*_^**U**^.

In contrast to 2DLDA, 2DMMC has the following advantages which makes it stable and efficient: (1) the objective of (9) increases monotonically through iterations; hence the convergence of 2DMMC is rigorously guaranteed [8]; (2) 2DMMC avoids computing inverse matrices in each iteration.

However, as bilateral projection based 2D feature extraction techniques, 2DMMC, 2DLDA, and GLRAM share such shortcomings: The iterations and alternations are time-consuming, and an arbitrary initial value of **V** cannot guarantee the global optimum.

## 3. Proposed Framework

Bilateral projection based 2D feature extraction techniques, such as 2DMMC, 2DLDA, and GLRAM, consider seeking transforms on both sides of image matrices, that is, both left and right projections are taken, but the computation of two-side projection matrices involves time-consuming iterations and alternations, and the initialization before iterations may lead to local optimum. In our study, in order to overcome forgoing shortcomings, we propose a framework that only takes right multiplication of each block to extract the inter-row spatial information. Our block-wise approach to face recognition, namely, Block-wise Two Dimensional Maximum Margin Criterion (B2D-MMC), is described as follows.

### 3.1. Block-Wise Model for Face Recognition

**Figure 1:**
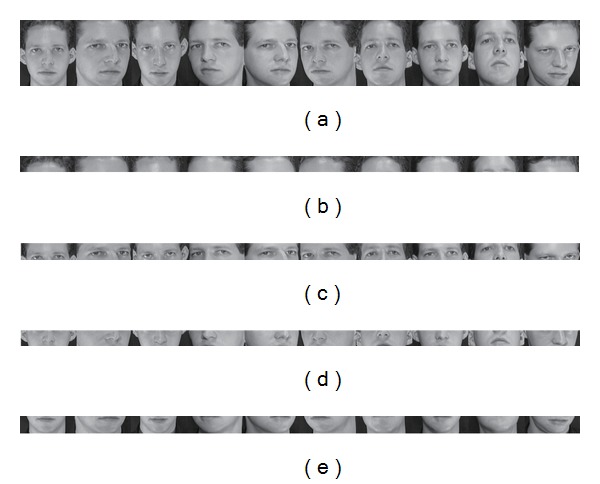
Images and their blocks of the first subject from ORL database: (a) 10 images of size 112 × 92; (b)–(e) 4 image blocks of size 28 × 92 for each image in (a).

Since we deal with images cropped either manually or by a face detection procedure, our block-wise model divides the face image into nonoverlapping groups of rows, which are called image blocks. Let **X** ∈ ℝ^*n*×*m*^ denote a face image, where *n*, *m* are the numbers of rows and columns of **X**, respectively. **X** is divided into *n*_*B*_ nonoverlapping image blocks **X**(*k*) ∈ ℝ^*r*_*B*_×*m*^, *k* = 1,2,…, *n*_*B*_, each including *r*_*B*_ rows of image **X**.  Figure 1 shows an example of image blocks. In the example, images of the first subject from the ORL database, which have the size of 112 × 92, are partitioned into four blocks of size 28 × 92, that is, *n* = 112, *m* = 92, *n*_*B*_ = 4, and *r*_*B*_ = 28.

For all sample images, the set of *k*th image blocks is referred to as the *k*th block set *𝔹𝕊*_*k*_, which spans a subspace referred to as the *k*th block manifold, *k* = 1,2,…, *n*_*B*_. The advocated B2D-MMC algorithm attempts to find a local subspace projection, that is, unilateral projection matrix, in each block set.

### 3.2. B2D-MMC

Considering a *C*-class problem, the *i*th class contains *N*_*i*_ training image matrices **X**_*j*_^(*i*)^ ∈ ℝ^*n*×*m*^, *j* = 1,2,…, *N*_*i*_, where **X**_*j*_^(*i*)^ is the *j*th training image in class *i*, *i* = 1,2,…, *C*, and *n*, *m* are the numbers of rows and columns of face images, respectively. Let *N* be the total number of training images, that is, *N* = ∑_*i*=1_^*C*^ *N*_*i*_.

As determined in Section 3.1, each image **X**_*j*_^(*i*)^ consists of *n*_*B*_ blocks, each block including *r*_*B*_ rows of the face image. Denoting the *k*th image block of **X**_*j*_^(*i*)^ as **X**_*j*_^(*i*)^(*k*) ∈ ℝ^*r*_*B*_×*m*^, *k* = 1,2,…, *n*_*B*_, we have

(14) $$\begin{array}{c} X_{j}^{\left(i\right)}=\left(\begin{array}{c} X_{j}^{\left(i\right)}\left(1\right)\\ X_{j}^{\left(i\right)}\left(2\right)\\ \vdots \\ X_{j}^{\left(i\right)}\left(n_{B}\right) \end{array}\right). \end{array}$$

Thus the *k*th block set *𝔹𝕊*_*k*_ can be formulated as

(15) 𝔹𝕊k={X1(1)(k),…,XN1(1)(k),X1(2)(k),…,XN2(2)(k),     …,X1(C)(k),…,XNC(C)(k)},        k=1,2,…,nB.

Also let **X**_*j*_^(*i*)^(*k*, *r*) ∈ ℝ^1×*m*^ be the *r*th row of **X**_*j*_^(*i*)^(*k*), *r* = 1,2,…, *r*_*B*_. Then we can write

(16) $$\begin{array}{c} X_{j}^{\left(i\right)}\left(k\right)=\left(\begin{array}{c} X_{j}^{\left(i\right)}\left(k,1\right)\\ X_{j}^{\left(i\right)}\left(k,2\right)\\ \vdots \\ X_{j}^{\left(i\right)}\left(k,r_{B}\right) \end{array}\right). \end{array}$$

For all training image matrices, the proposed B2D-MMC aims to find *n*_*B*_ orthogonal right-side projection matrices, one for each image block set; that is, given a desired dimensionality *l*, to find **V**(*k*) ∈ ℝ^*m*×*l*^ for the *k*th block set *𝔹𝕊*_*k*_, mapping the *k*th image block **X**_*j*_^(*i*)^(*k*) ∈ ℝ^*r*_*B*_×*m*^ to **Y**_*j*_^(*i*)^(*k*) ∈ ℝ^*r*_*B*_×*l*^, such that

(17) $$\begin{array}{c} Y_{j}^{\left(i\right)}\left(k\right)=X_{j}^{\left(i\right)}\left(k\right)V\left(k\right),\;k=1,2,\ldots ,n_{B}. \end{array}$$

And we use the following **Y**_*j*_^(*i*)^ ∈ ℝ^*n*×*l*^ as the feature of image **X**_*j*_^(*i*)^ for training:

(18) $$\begin{array}{c} Y_{j}^{\left(i\right)}=\left(\begin{array}{c} Y_{j}^{\left(i\right)}\left(1\right)\\ Y_{j}^{\left(i\right)}\left(2\right)\\ \vdots \\ Y_{j}^{\left(i\right)}\left(n_{B}\right) \end{array}\right)\;i=1,2,\ldots ,C,\;\;j=1,2,\ldots ,N_{i}. \end{array}$$

For classification, features of testing images are stacked by subfeatures in the same form as above.

The following shows how to find the *n*_*B*_ projection matrices **V**(*k*), *k* = 1,2,…, *n*_*B*_. Let **M**_*i*_(*k*) ∈ ℝ^*r*_*B*_×*m*^ and **M**(*k*) ∈ ℝ^*r*_*B*_×*m*^ denote the mean of the *k*th image blocks in the *i*th class and the mean of the *k*th block set *𝔹𝕊*_*k*_, respectively, as follows

(19) $$\begin{array}{c} M_{i}\left(k\right)=\frac{1}{N_{i}}\overset{N_{i}}{\underset{j=1}{\sum }}X_{j}^{\left(i\right)}\left(k\right),\\ M\left(k\right)=\frac{1}{N}\overset{C}{\underset{i=1}{\sum }}\overset{N_{i}}{\underset{j=1}{\sum }}X_{j}^{\left(i\right)}\left(k\right). \end{array}$$

Also let **m**_*i*_(*k*, *r*) ∈ ℝ^1×*m*^ and **m**(*k*, *r*) ∈ ℝ^1×*m*^ be the *r*th row of **M**_*i*_(*k*) and **M**(*k*), respectively, *r* = 1,2,…, *r*_*B*_. Then we have

(20) $$\begin{array}{c} m_{i}\left(k,r\right)=\frac{1}{N_{i}}\overset{N_{i}}{\underset{j=1}{\sum }}X_{j}^{\left(i\right)}\left(k,r\right),\\ m\left(k,r\right)=\frac{1}{N}\overset{C}{\underset{i=1}{\sum }}\overset{N_{i}}{\underset{j=1}{\sum }}X_{j}^{\left(i\right)}\left(k,r\right). \end{array}$$

Let us define the between-class block scatter matrix **S**_*b*_(*k*) and within-class block scatter matrix **S**_*w*_(*k*) of the *k*th block set *𝔹𝕊*_*k*_ respectively as follows

(21) $$\begin{array}{c} S_{b}\left(k\right)=\overset{C}{\underset{i=1}{\sum }}N_{i}{\left(M_{i}\left(k\right)-M\left(k\right)\right)}^{T}\left(M_{i}\left(k\right)-M\left(k\right)\right),\\ S_{w}\left(k\right)=\overset{C}{\underset{i=1}{\sum }}\overset{N_{i}}{\underset{j=1}{\sum }}{\left(X_{j}^{\left(i\right)}\left(k\right)-M_{i}\left(k\right)\right)}^{T}\left(X_{j}^{\left(i\right)}\left(k\right)-M_{i}\left(k\right)\right), \end{array}$$

*k* = 1,2,…, *n*_*B*_. It is easy to verify that **S**_*b*_(*k*) and **S**_*w*_(*k*) are two *m* × *m* nonnegative definite matrices from their definitions.

In the low dimensional space resulting from the *k*th linear transformation **V**(*k*), as in 2DMMC [8], we adopt the Frobenius norm ||·||_*F*_ [10] as the metric of matrices, that is, ||**A**||_*F*_^2^ = tr⁡(**A** **A** ^*T*^) = tr⁡(**A** ^*T*^ **A**) for any matrix **A**. Under this metric, the projected between-class block scatter ${\tilde{S}}_{b}(k)$ and projected within-class block scatter ${\tilde{S}}_{w}(k)$ can be respectively defined as follows

(22) S~b(k)=∑i=1CNi||(Mi(k)−M(k))V(k)||F2=tr⁡(∑i=1CNiV(k)T(Mi(k)−M(k))T  ×(Mi(k)−M(k))V(k)),S~w(k)=∑i=1C∑j=1Ni||(Xj(i)(k)−Mi(k))V(k)||F2=tr⁡(∑i=1C∑j=1NiV(k)T(Xj(i)(k)−Mi(k))T  ×(Xj(i)(k)−Mi(k))V(k)),           k=1,2,…,nB.

The proposed B2D-MMC finds the orthogonal projection matrix **V**(*k*) for the *k*th block set *𝔹𝕊*_*k*_ by the following optimization:

(23) max⁡V(k) S~b(k)−μkS~w(k),s.t.  V(k)TV(k)=I,

where *μ*_*k*_ is a weighted parameter, *k* = 1,2,…, *n*_*B*_.

In order to compute **V**(*k*), *k* = 1,2,…, *n*_*B*_, comparing (21) with (22), the following relation is held:

(24) $$\begin{array}{c} {\tilde{S}}_{b}\left(k\right)-\mu_{k}{\tilde{S}}_{w}\left(k\right)=\mathrm{tr}\left(V{\left(k\right)}^{T}\left(S_{b}\left(k\right)-\mu_{k}S_{w}\left(k\right)\right)V\left(k\right)\right). \end{array}$$

Thus the optimal **V**(*k*) can be computed by solving a eigen-decomposition on *m* × *m* matrix **S**_*b*_(*k*) − *μ*_*k*_ **S**_*w*_(*k*); that is,

(25) $$\begin{array}{c} V\left(k\right)=\left\{v_{1}\left(k\right),v_{2}\left(k\right),\ldots ,v_{l}\left(k\right)\right\}, \end{array}$$

where *m* × 1 vector **v**_*i*_(*k*) is the eigenvector corresponding to the *i*th largest eigenvalue of **S**_*b*_(*k*) − *μ*_*k*_ **S**_*w*_(*k*), *i* = 1,2,…, *l*, *k* = 1,2,…, *n*_*B*_.

From the description above, it is easy to see that our B2D-MMC has the following two advantages compared with 2DMMC [8]. 

*(a) Computational Complexity. *B2D-MMC seeks a closed form solution of unilateral projection matrix for each block set instead of finding iterative solutions of two projection matrices for the entire image matrix, avoiding iterations and alternations as in 2DMMC, which saves the computational effort. 

*(b) Locality. *Based on the block-wise model, B2D-MMC learns local characteristics of input image by dividing the face image into non-overlapping image blocks. Expectedly, distribution of data is much less complex inside these block manifolds.

### 3.3. Algorithm Design

Based on the analysis above, our B2D-MMC algorithm is designed as in Algorithm 1.

In our experiments reported in Section 4, the parameter *μ*_*k*_ is set as tr⁡**S**_*b*_(*k*)/tr⁡**S**_*w*_(*k*) according to [11], *k* = 1,2,…, *n*_*B*_.

### 3.4. Computational Complexity Analysis

Most of the algorithms involve computations scale to *O*(*h* ^3^) for eigen-decomposition of an *h* × *h* matrix [10]. The eigen-decomposition of the scatter matrices in B2D-MMC amounts to a complexity of *O*(*m* ^3^). However, as reviewed in Section 2, in 2DLDA and 2DMMC, the scatter matrices in each iteration are of size *n* × *n*, so the overall computation complexity of 2DMMC is *O*(*tn* ^3^), where *t* is the number of iterations. Obviously we can expect that *O*(*m* ^3^) is smaller than *O*(*tn* ^3^) when *t* is considerable.

## 4. Experiments

In this section, to investigate the performance of the proposed B2D-MMC for face recognition, we compare our method with PCA [1], LDA [2], MMC [3], GLRAM [6], 2DLDA [7], and 2DMMC [8], in both accuracy and efficiency. Furthermore, the effect of image block size on recognition results is investigated.

### 4.1. Performance Comparison

#### 4.1.1. Face Datasets

In our experiment, we use two standard face recognition databases which are widely used as bench mark datasets in feature extraction literature.

**Figure 2:**
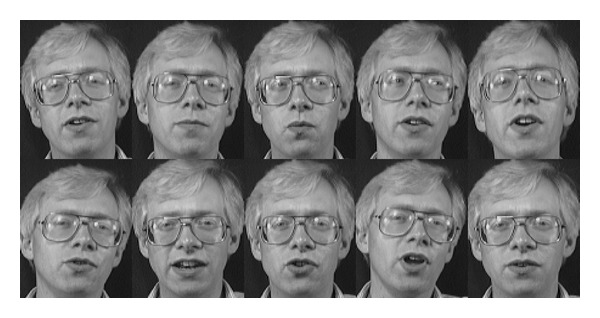
Images of one person from the ORL face database.

*The ORL Face Database. *There are ten images for each of the 40 human subjects, which were taken at different times, varying the lighting, facial expressions and facial details. Images from one subject are shown in Figure 2. The original images (with 256 gray levels) have size 92 × 112, which are resized to 32 × 32 for efficiency. 

**Figure 3:**
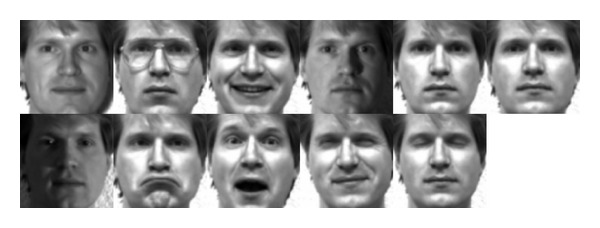
Images of one person from the Yale face database.

*The Yale Face Database. *It contains 11 gray scale images for each of the 15 individuals. The images demonstrate variations in lighting condition, facial expression, and with/without glasses. Images from one subject are shown in Figure 3. In our experiment, the images were also resized to 32 × 32.

#### 4.1.2. Parameter Settings for B2D-MMC

For each individual, TN = 2,3, 4 images were randomly selected as training samples, and the rest were used for testing. The training set was used to learn *n*_*B*_ = 4 subspaces, each for one block set. Thus the size of the block set is 8 × 32. Features of images for classification were stacked by sub-features in the form of (18), and the recognition was performed by Nearest Neighbor Classifier, with the Frobenius norm as the similarity metric. Since the training set was randomly chosen, we repeated each experiment 20 times and calculated the average recognition accuracy. In general, the recognition rate varies with *l*, that is, the number of columns of the feature (projected image). We set *l* to the corresponding dimensionality when the best performance was obtained by 2DMMC [8].

#### 4.1.3. Comparison on Classification Accuracy

Tables 1 and 2 show the experimental results of the proposed B2D-MMC on the two databases, respectively, with the *best* results of PCA, LDA, MMC, GLRAM, 2DLDA, and 2DMMC referred from [8] for comparison. For all the methods, the value in each entry represents the average recognition accuracy of 20 independent trials, and the number in brackets is the corresponding projection dimensionality.

Since the value of dimensionality *l*, which corresponds to the best performance obtained by 2DMMC, is not necessarily the best choice for our B2D-MMC, it is clear that B2D-MMC outperforms 2DMMC and the other feature extraction methods on both of the two data sets.

#### 4.1.4. Comparison on Efficiency

In this subsection, B2D-MMC is compared with 2DMMC in computational efficiency. We take the ORL and the Yale datasets where TN = 2 for example; that is, two training samples are randomly selected for each subject.

For 2DMMC, we record the training time in the following way: taking the entries in Tables 1 and 2 as the best classification accuracies, that is, 78.75% as the best on the ORL and 54.37% as the best on the Yale dataset, the iteration of training process stops if the difference between the obtained classification accuracy and the best classification accuracy is smaller than 0.1%. And the projection dimensionality of the training process is set to the corresponding value of the best classification, that is, 12 × 12 for the ORL and 6 × 6 for the Yale dataset.

**Figure 4:**
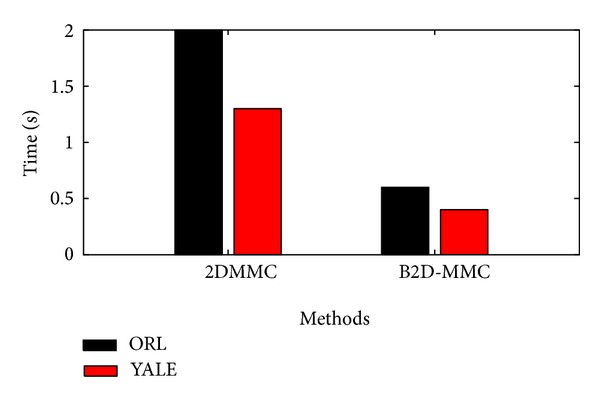
Training time of two methods on ORL and Yale datasets with TN = 2.

The average training time of B2D-MMC and 2DMMC, over 20 independent runs on a typical laptop using MATLAB, is shown in Figure 4. It can be seen that B2D-MMC is more efficient than 2DMMC, both on the ORL and on the Yale dataset. This conforms to the complexity analysis in Section 3.4. This must be because that, unlike 2DMMC which is to find iterative solutions of two projection matrices for the entire image matrix, B2D-MMC seeks a closed form solution of one-side projection matrix for each block set, avoiding iterations and alternations as in 2DMMC, which decreases the computational load.

### 4.2. Effect of Number of Blocks on Recognition Results

**Figure 5:**
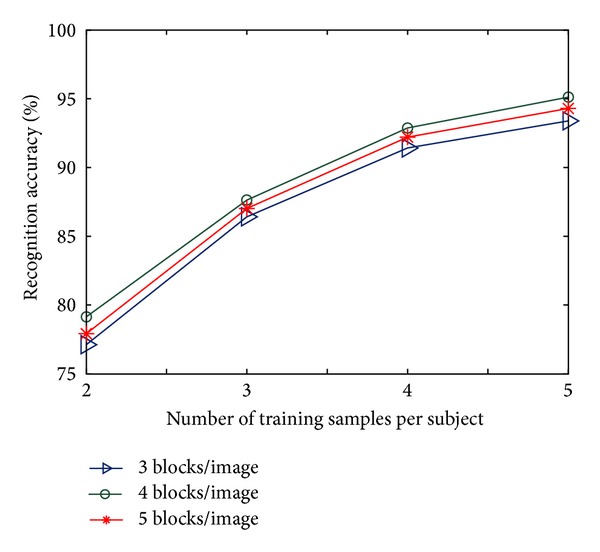
Recognition performance on the ORL dataset for different number of blocks per image.

**Figure 6:**
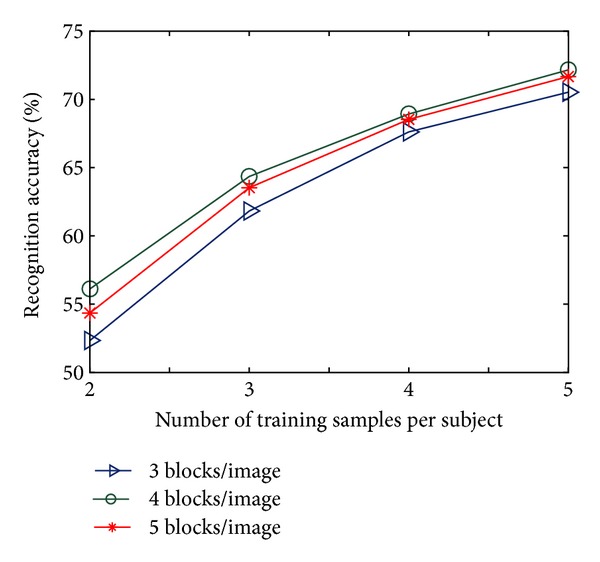
Recognition performance on the Yale dataset for different number of blocks per image.

The proposed B2D-MMC has been applied on the ORL and the Yale datasets with the same settings as in Section 4.1 but for three different values of *n*_*B*_, namely 3, 4, and 5. Results, shown in Figures 5 and 6, reveal that the performance of B2D-MMC achieves optimum when *n*_*B*_ takes an appropriate value, for example, *n*_B_* = 4, and neither raising nor reducing the value of *n*_B_* degrades the performance of B2D-MMC. It can be interpreted as follows. An increase in the number of blocks per image helps to learn more local characteristics; however, a decrease in the number helps to utilize more global characteristics. The optimal recognition performance results from the tradeoff between local and global information.

## 5. Conclusions

This paper proposed a novel framework to extract discriminating features directly from 2D face images. The proposed B2D-MMC introduces a block-wise model for face recognition, performing one-side subspace projection inside each block manifold, in which a block is close to those belonging to the same class but far from those belonging to different classes. The unilateral projection and the block-wise learning avoid iterations and alternations as in current bilateral projection based two-dimensional feature extraction approaches, and have advantages in complexity and locality.

Computational complexity analysis shows that B2D-MMC consumes less time than 2DLDA and 2DMMC when the number of iterations for the latter is considerable. Performance comparison experiments on the ORL and the Yale datasets illustrate that B2D-MMC is more effective and efficient than current bilateral projection based two-dimensional feature extraction techniques.

## Figures and Tables

**Algorithm 1:**
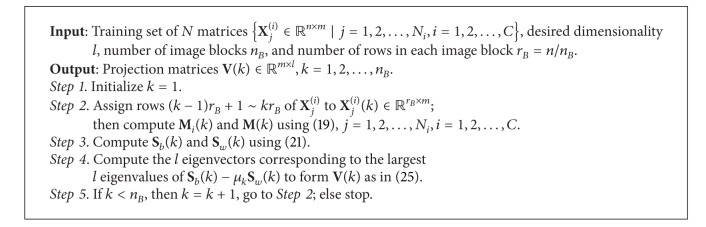


**Table 1:** Face recognition accuracies of different methods on the ORL database. TN means number of training samples per subject, and the number in brackets is the corresponding projection dimensionality. The bold value means the highest accuracy among all the methods.

Method	TN = 2	TN = 3	TN = 4
PCA	70.67% (79)	78.88% (118)	84.21% (152)
LDA	72.80% (25)	83.79% (39)	90.13% (39)
MMC	77.97% (39)	86.32% (39)	91.63% (39)
GLRAM	71.30% (17 × 17)	79.84% (11 × 11)	84.73% (16 × 16)
2DLDA	78.13% (11 × 11)	86.79% (16 × 16)	92.08% (15 × 15)
2DMMC	78.75% (12 × 12)	87.50% (10 × 10)	**92.92**% (8 × 8)
B2D-MMC	**79.14**% (*l* = 12)	** 87.63**% (*l* = 10)	92.88% (*l* = 8)

**Table 2:** Face recognition accuracies of different methods on the Yale database. TN means number of training samples per subject, and the number in brackets is the corresponding projection dimensionality. The bold value means the highest accuracy among all the methods.

Method	TN = 2	TN = 3	TN = 4
PCA	46.04% (29)	49.96% (44)	55.67% (58)
LDA	42.81% (11)	60.33% (14)	68.10% (13)
MMC	52.37% (14)	61.83% (14)	67.95% (15)
GLRAM	49.33% (6 × 6)	54.17% (6 × 6)	57.76% (5 × 5)
2DLDA	44.37% (7 × 7)	59.71% (5 × 5)	68.71% (5 × 5)
2DMMC	54.37% (6 × 6)	63.50% (9 × 9)	68.86% (15 × 15)
B2D-MMC	**56.11**% (*l* = 6)	** 64.35**% (*l* = 9)	** 68.92**% (*l* = 15)
